# Ten tips for the diagnosis and management of nephrotic syndrome

**DOI:** 10.1093/ckj/sfag268

**Published:** 2026-08-12

**Authors:** Ilay Berke, Annette Bruchfeld, Eleni Frangou, Sarah M Moran, Luis F Quintana, Kate I Stevens, Y K Onno Teng, Stefanie Steiger, Andreas Kronbichler, Safak Mirioglu

**Affiliations:** Department of Internal Medicine IV, Nephrology and Hypertension, Medical University Innsbruck, Innsbruck, Austria; Department of Health, Medicine and Caring Sciences, Linköping University, Linköping, Sweden; Department of Renal Medicine, Karolinska University Hospital and CLINTEC Karolinska Institutet, Stockholm, Sweden; Department of Nephrology, Limassol General Hospital, State Health Service Organization, Limassol, Cyprus; University of Nicosia Medical School, Nicosia, Cyprus; Medical School, National and Kapodistrian University of Athens, Athens, Greece; Cork University Hospital, University College Cork, Cork, Republic of Ireland; National Reference Centre on Complex Glomerular Disease (CSUR), Nephrology Department, Hospital Clinic de Barcelona, IDIBAPS, University of Barcelona, Spain; Glasgow Renal and Transplant Unit, Queen Elizabeth University Hospital, Glasgow, UK; Center of Expertise for Lupus-, Vasculitis- and Complement-Mediated Systemic Diseases, Department of Internal Medicine – Nephrology Section, Leiden University Medical Center, Leiden, The Netherlands; Division of Nephrology, Department of Internal Medicine IV, Hospital of the Ludwig Maximilians University of Munich, Munich, Germany; Department of Internal Medicine IV, Nephrology and Hypertension, Medical University Innsbruck, Innsbruck, Austria; Division of Nephrology, Department of Internal Medicine, Istanbul Faculty of Medicine, Istanbul University, Istanbul, Turkey; Department of Immunology, Aziz Sancar Institute of Experimental Medicine, Istanbul University, Istanbul, Turkey

**Keywords:** cardiovascular risk, genetic testing, immunosuppression, kidney biopsy, nephrotic syndrome

## Abstract

Nephrotic syndrome (NS) encompasses a heterogeneous group of glomerular diseases characterized by heavy proteinuria, hypoalbuminemia, edema, and multiple systemic complications. Despite substantial advances in diagnostic techniques and targeted therapies, the management of NS remains challenging in clinical practice. In addition to complex treatment decisions, clinicians must address diagnostic uncertainty, recognize secondary causes, and prevent potentially serious complications including thrombosis, infections, cardiovascular disease, and progressive kidney dysfunction. Adverse outcomes in NS frequently arise not only from inappropriate management but also from overlooked steps in diagnosis, monitoring, and long-term care. Here, we present 10 practical tips to improve clinical decision-making in the diagnosis and management of NS. Key positive strategies include appropriate use of kidney biopsy and modern immunopathologic techniques, integration of immunologic biomarkers such as anti-phospholipase A2 receptor antibodies in the evaluation of membranous nephropathy, systematic exclusion of secondary causes, and comprehensive supportive care including blood pressure control, antiproteinuric therapy, and thrombosis prophylaxis in high-risk patients. In addition, evidence-based use of immunosuppressive therapies (IST) tailored to specific disease entities is emphasized. Equally important are commonly overlooked pitfalls that may compromise outcomes, including delayed genetic testing in steroid-resistant disease, prolonged ineffective immunosuppression, inadequate post-transplant surveillance for recurrence, under-recognition of cardiovascular risk, and failure to prevent complications associated with IST. Highlighting these practical considerations may help increase awareness of common pitfalls and support more attentive clinical management of patients with NS.

**Figure:**
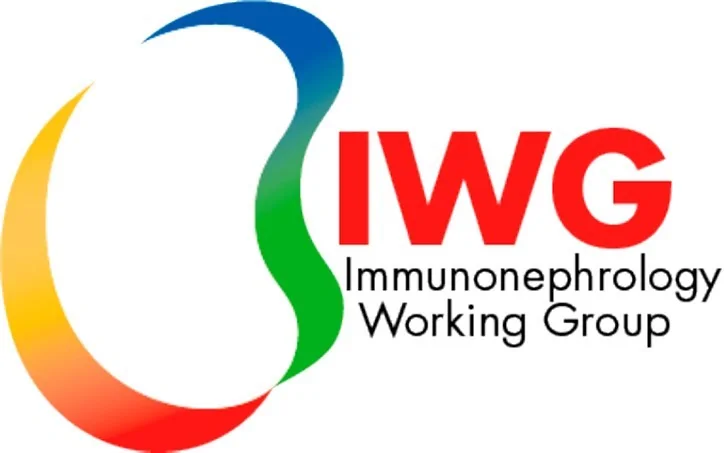


## INTRODUCTION

Nephrotic syndrome (NS) represents a heterogeneous group of glomerular diseases characterized by heavy proteinuria, hypoalbuminemia, edema, and metabolic complications. Despite major advances in diagnostic approaches and targeted therapies, the management of NS remains complex. Clinicians must simultaneously address diagnostic uncertainty, disease-specific treatment decisions, and the prevention of systemic complications such as thrombosis, infections, cardiovascular morbidities, and progressive kidney failure. In daily clinical practice, errors often arise not only from what is done incorrectly but also from what is overlooked.

Traditional reviews of NS management tend to focus on recommended diagnostic steps and treatment algorithms. However, it is equally important to highlight common pitfalls which may compromise patient outcomes. Delayed or incomplete diagnostic work-up, under-recognition of secondary causes, insufficient monitoring for complications, and inadequate preventive strategies can all lead to substantial morbidity [1, 2]. Importantly, many of these risks emerge across different etiologies of NS and can occur throughout the disease course, from initial diagnosis to long-term follow-up.

**Figure 1:**
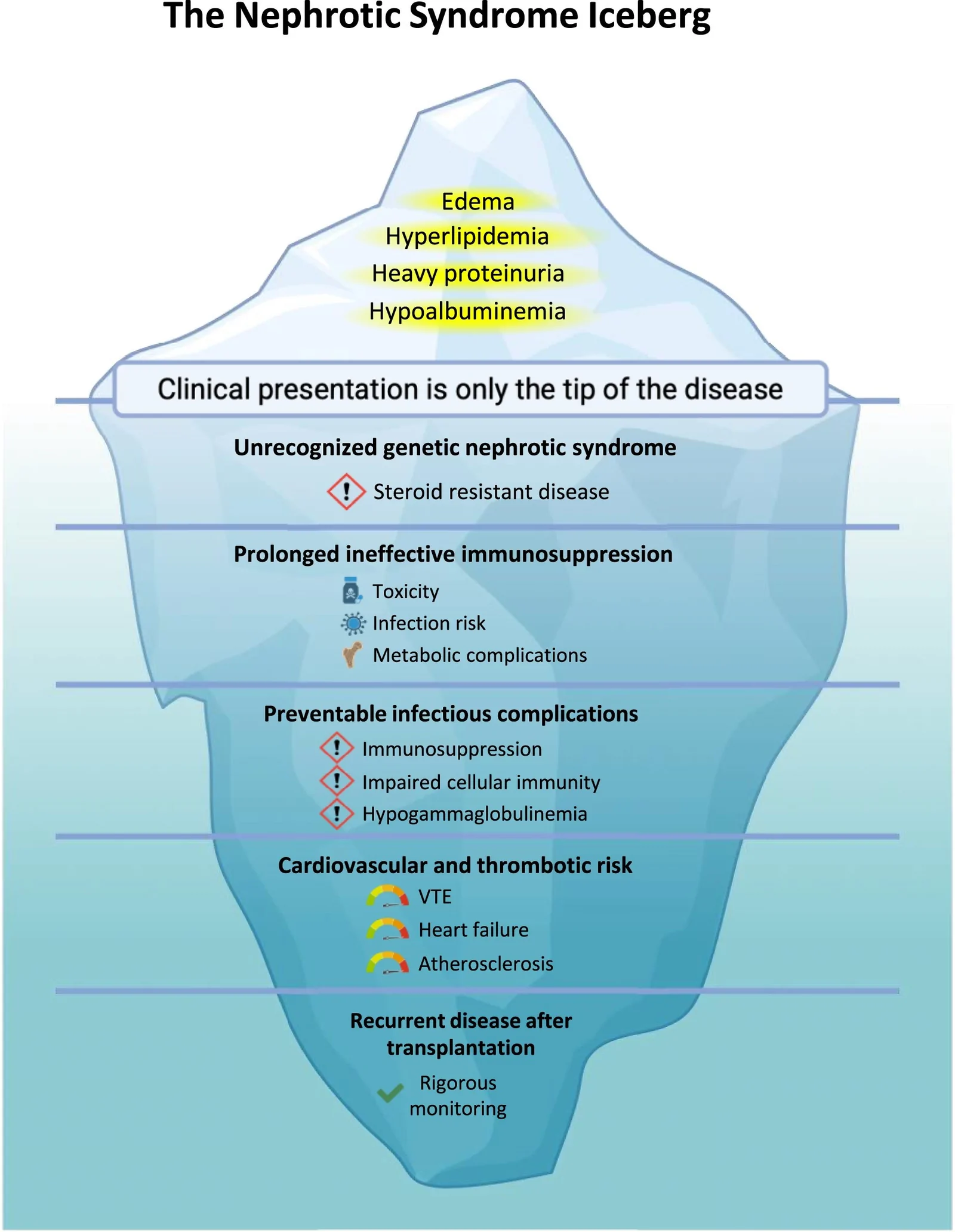
Nephrotic syndrome can be viewed as an iceberg, where the clinical presentation represents only the visible tip of the disease and requires careful management. Steroid resistance may indicate an underlying genetic etiology. Physicians should remain vigilant for complications arising from both the disease itself and IST. Careful monitoring is also essential to detect recurrence after transplantation. VTE: venous thromboembolism.

This article presents a practical framework of “positive and negative tips” in the diagnosis and management of NS, focusing on critical mistakes that clinicians should avoid (Tables 1 and 2). We emphasize areas where vigilance is essential and, as illustrated in Fig. 1, visualize this framework as an iceberg, since much of the disease burden lies beneath the visible clinical presentation. By addressing these frequently overlooked aspects of care, clinicians may improve diagnostic precision, optimize treatment decisions, and reduce preventable complications in patients with NS.

**Table 1:** Positive tips for the diagnosis and management of nephrotic syndrome.

Topic	Recommendation	Literature
P1 — Kidney biopsy and pathology	- In patients with diagnostic uncertainty^a^, perform a kidney biopsy to identify the underlying disease and guide treatment. - Assess the degree of foot process effacement by electron microscopy when available.	[3–22]
P2 — Immunologic testing	- Test for antibodies against phospholipase A2 receptor (PLA2R), other MN antigens, and podocyte structures (if available), to identify autoimmune causes and guide immunological monitoring.	[13, 23–32]
P3 — Exclude secondary causes	- Obtain a thorough medical history and conduct appropriate investigations to rule out secondary causes.	[33–39]
P4 — Supportive and prophylactic management	- Monitor blood pressure, salt intake, lipid profile, and edema regularly. - Start prophylactic anticoagulation in patients with membranous nephropathy or with persistent nephrosis at high risk of thromboembolic events.	[1, 13, 40–44]
P5 — Specific IST	- Use glucocorticoids as first-line treatment for primary podocytopathies. - Consider B-cell-depleting agents or calcineurin inhibitors in relapsing or steroid-dependent/resistant cases. - For primary membranous nephropathy, use rituximab in patients without high risk of progression; reserve cyclophosphamide plus glucocorticoids for those with severe kidney function impairment or life-threatening complications. - Newer B-cell-depleting agents are suggested in resistant patients with primary podocytopathies and MN.	[10, 13, 33, 47–59]

^a^In the absence of diabetes or secondary causes, positive anti-PLA2R antibodies in the serum strongly predict membranous nephropathy in patients with preserved glomerular filtration rate, eliminating the need for kidney biopsy [19].

**Table 2:** Negative tips for the diagnosis and management of nephrotic syndrome.

Topic	Recommendation	Literature
N1 — Do not delay genetic testing	- Do not postpone genetic testing in primary nephrotic syndrome when remission is not achieved.	[60–68]
N2 — Do not prolong ineffective immunosuppression	- Avoid continuing IST without clinical response in order to minimize toxicity and infection risk.	[27, 69, 72–75]
N3 — Do not miss post-transplant monitoring	- Do not neglect post-transplant surveillance for early recurrence in patients with primary NS.	[76–82]
N4 — Do not ignore cardiovascular risk	- Do not overlook cardiovascular risk factors such as persistent hypoalbuminemia, dyslipidemia, and reduced eGFR. Cardiovascular assessment should be integrated into long-term care.	[1, 83, 84]
N5 — Do not overlook preventable infectious complications in patients receiving IST	- Screen for latent infections and give antimicrobial prophylaxis, if indicated.	[2, 85–93]

### P1 — Kidney biopsy and pathology

#### In patients with diagnostic uncertainty, perform a kidney biopsy to identify the underlying disease and guide treatment. Assess the degree of foot process effacement by electron microscopy when available.

We have witnessed significant advances in understanding the pathological mechanisms and diagnosing NS over the last few decades. Nevertheless, a kidney biopsy remains the gold standard for assessing the precise pattern of injury and is, overall, a safe procedure [3, 4]. The specimen is usually examined with light microscopy, immunofluorescence or immunohistochemistry, and electron microscopy (when available) in daily practice [5]. In cases where frozen tissue is not available or lacks glomeruli, immunofluorescence can be performed on paraffin tissue after antigen retrieval (paraffin immunofluorescence) [6]. This salvage technique delivers excellent results in immune complex-mediated and monoclonal protein-associated disorders. Notably, immunohistology has become an important part of the examination, providing information on antigens or biomarkers associated with membranous nephropathy (MN), the typing of amyloidosis, and the accurate diagnosis of fibrillary glomerulonephritis, as well as IgG subclasses [7]. Very recently, apolipoprotein E staining has shown very strong potential to obviate the need for electron microscopy to characterize the subtypes of C3 glomerulopathy [8, 9]. Focal segmental glomerulosclerosis (FSGS) is a histopathological lesion that can be caused by autoantibodies (primary), pathological genetic variants, or secondary insults [10]. The extent of the lesion of the podocyte processes by electron microscopy could reveal the underlying cause. Primary FSGS is expected to have a foot process effacement of at least 80% (diffuse effacement) [11, 12]. However, genetic variants can also cause diffuse effacement, and cases of presumed primary FSGS with lower degrees of effacement have been reported [10, 13]. Beyond its diagnostic use, examination of the kidney tissue can provide crucial information on prognosis. The Oxford classification is a great example that has been incorporated into the International IgA Nephropathy Prediction Tool [14, 15]. Notably, a histologic scoring index is also used to assess C3 glomerulopathy [16–18].

In certain circumstances, immunologic testing may remove the need for a kidney biopsy altogether. The best validated example is MN, in which circulating anti-phospholipase A2 receptor (anti-PLA2R) antibodies have high diagnostic specificity. In a landmark validation study, it was demonstrated that patients with nephrotic-range proteinuria, positive serum anti-PLA2R antibodies detected by enzyme-linked immunosorbent assay (ELISA) or indirect immunofluorescence, and no clinical or serological evidence of secondary causes had an ∼100% positive predictive value for primary MN, supporting a non-invasive diagnosis in this specific clinical setting [19]. Therefore, in patients who meet these criteria and have preserved kidney function, kidney biopsy may be safely deferred without compromising diagnostic accuracy.

However, the availability of anti-PLA2R testing does not eliminate the need for a kidney biopsy in all patients with suspected MN. Histologic evaluation remains particularly important in patients with diabetes, in whom concomitant diabetic kidney disease may be present and false-positive anti-PLA2R ELISA results have been reported [20]. Biopsy is also warranted in patients with atypical features, including rapidly declining kidney function or active urinary sediment, as well as in those with findings suggestive of secondary MN such as active systemic autoimmune disease, hepatitis B or C infection, or suspected malignancy. Furthermore, a biopsy should be considered when there is discordance between anti-PLA2R antibody titers and disease severity or clinical course. Tissue examination remains indispensable in PLA2R-negative MN, where antigen-based classification using markers such as thrombospondin type-1 domain-containing 7A (THSD7A), neural epidermal growth factor-like 1 (NELL1), exostosin 1/exostosin 2 (EXT1/EXT2), neural cell adhesion molecule 1 (NCAM1), semaphorin-3B (SEMA3B), and protocadherin-7 (PCDH7) can provide important diagnostic and pathogenetic information [21]. Thus, while serologic testing has enabled a non-invasive diagnosis in selected patients, kidney biopsy continues to play a critical role in diabetic patients and in atypical, secondary, or antigen-negative forms of MN.

Anti-nephrin antibodies have a similar potential, but they are not routinely used yet due to a lack of standardized commercial testing options [22].

### P2 — Immunologic testing

#### Test for antibodies against PLA2R, other MN antigens, and podocyte structures (if available) to identify autoimmune causes and guide immunological monitoring.

Conventional immunologic biomarkers, such as antinuclear antibodies and serum levels of C3 and C4, have long been helpful in the differential diagnosis of NS. However, immunologic testing has become an integral part of the process following the discovery of anti-PLA2R autoantibodies [23]. Anti-PLA2R antibodies appear before the onset of NS, and immunologic remission is followed by clinical remission [24]. Serum levels of such antibodies are a reliable biomarker for disease activity, and standardized testing with ELISAs or indirect immunofluorescence tests is commercially available [13]. Antibodies directed against THSD7A can also be detected in serum by indirect immunofluorescence tests. However, these antibodies are quite rare in patients with MN (2%) [21]. Unfortunately, current commercial testing methods for sera do not yield reliable results for other, more recently uncovered MN antigens. A study reported high specificity but low sensitivity of an experimental ELISA for anti-NELL1 antibodies [25]. Kidney biopsy and immunohistology are still needed for diagnosis in most PLA2R-negative patients.

Anti-nephrin antibodies were discovered in late 2021 [26]. Subsequent studies have established nephrin as a major autoantigen in podocytopathies, with anti-nephrin antibodies detected in up to 90% of treatment-naïve children with minimal change disease (MCD) and in ∼30%–50% of affected adults [27]. Importantly, serum antibody levels correlate with disease activity and decline during remission, providing the first robust serological biomarker for a disease that has traditionally been diagnosed by exclusion and kidney biopsy.

This discovery has redefined the framework of podocytopathies and led to the concept of antibody-mediated podocytopathies [28, 29]. In this model, a subset of patients with MCD and FSGS may have an antibody-driven disease process that is distinct from genetic or secondary forms. Notably, primary MN itself is a mechanistically antibody-mediated disease directed against podocyte antigens, but it is classified separately from MCD and FSGS. In FSGS, anti-nephrin antibody positivity may help identify patients more likely to benefit from immunosuppressive therapies (IST), including B-cell-depleting strategies, whereas antibody-negative patients may warrant earlier genetic evaluation and investigation of secondary causes. Thus, anti-nephrin testing has the potential not only to refine diagnosis but also to guide therapeutic decision-making. Emerging evidence also suggests a potential role for anti-nephrin antibodies in predicting post-transplant recurrence of FSGS, further supporting their value as biomarkers across multiple stages of disease management [30–32]. However, their low titers in the serum have created challenges against standardized commercial testing so far, and most centers currently lack the ability to test [22]. Therefore, at present, without a standardized, validated commercial assay, these applications of anti-nephrin antibody testing remain promising but not yet actionable in routine practice. Autoantibodies against other components of the slit diaphragm, such as podocin and Kirrel1, have also been reported [28].

### P3 — Exclude secondary causes

#### Obtain a thorough medical history and conduct appropriate investigations to rule out secondary causes.

Investigation of potential secondary causes is a critical step in evaluating nephrotic syndrome and should be guided by the underlying histopathological pattern. A focused diagnostic work-up includes screening for metabolic disorders, infections, autoimmune diseases, monoclonal gammopathies, genetic causes, medications, and malignancies, with additional investigations tailored to the suspected glomerular disease.

**Figure 2:**
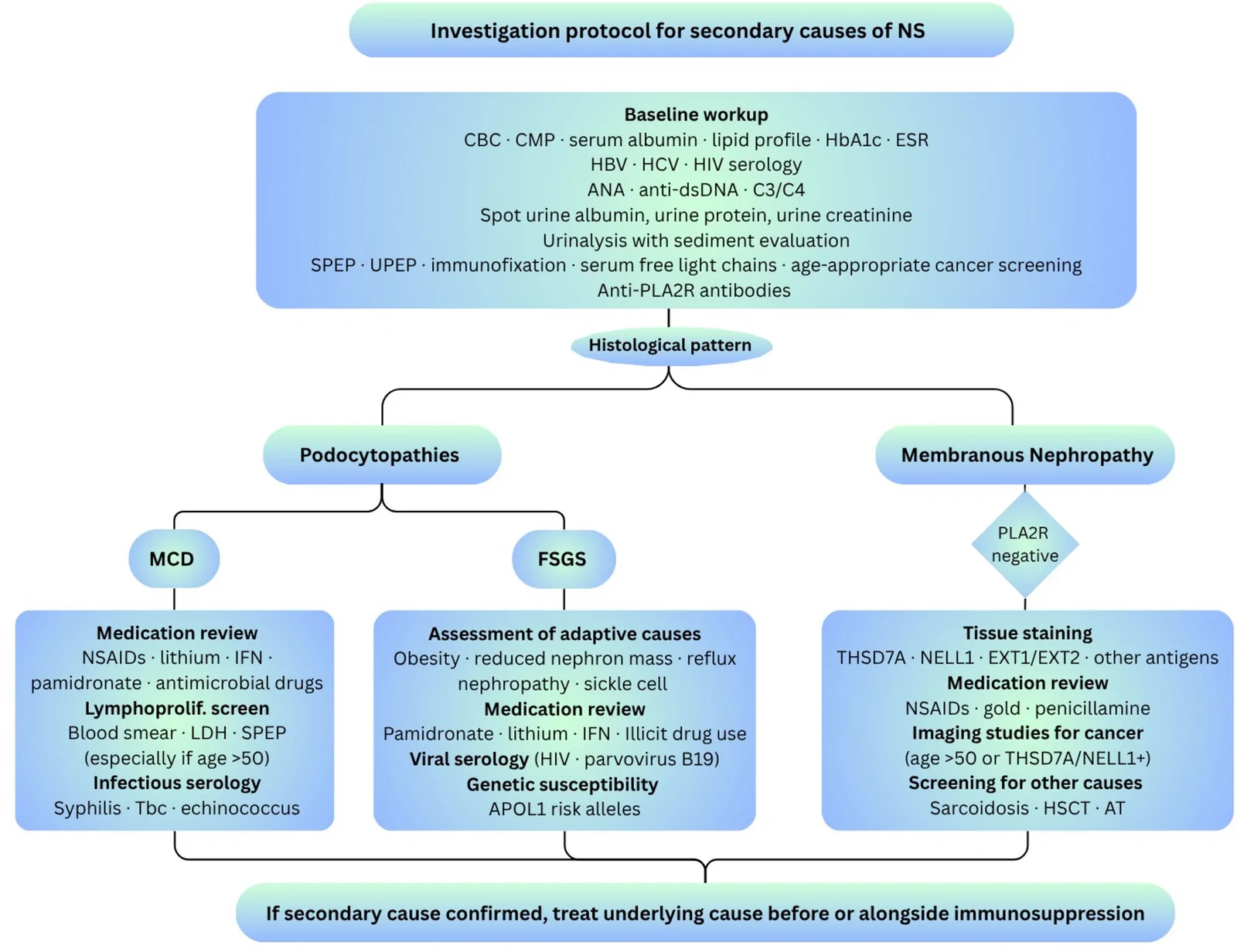
A minimal investigation protocol for secondary causes of nephrotic syndrome is presented. The branches reflect the biopsy-based diagnostic work-up pattern rather than disease nosology. NS, nephrotic syndrome; CBC, complete blood count; CMP, comprehensive metabolic panel; HbA1c, glycosylated hemoglobin; ESR, erythrocyte sedimentation rate; HBV, hepatitis B virus; HCV, hepatitis C virus; HIV, human immunodeficiency virus; SPEP, serum protein electrophoresis; UPEP, urine protein electrophoresis; PLA2R, phospholipase A2 receptor; MCD, minimal change disease; FSGS, focal segmental glomerulosclerosis; NSAID, non-steroidal anti-inflammatory drug; IFN, interferon; LDH, lactate dehydrogenase; Tbc, tuberculosis; THSD7A, thrombospondin type-1 domain containing 7A; NELL1, neural epidermal growth factor-like 1; EXT1/2, exostosin 1/2; HSCT, hematopoietic stem cell transplantation; AT, autoimmune thyroiditis.

An imbalance between increased metabolic demand (e.g. obesity and diabetes) and the limits of podocyte physiology can result in secondary FSGS. Medical history is particularly important in identifying adaptive causes, low nephron endowment, nephron loss, and exposure to potentially causative medications. Infectious agents and pathogenic variants in podocyte genes, as well as apolipoprotein L1 (APOL1) risk alleles, may also underlie podocytopathies [33]. Malignancy screening should be considered in selected patients, especially those with MN, as THSD7A- and NELL1-associated disease has been linked to an increased risk of cancer [34]. Lymphoproliferative disorders, although uncommon, may underlie some cases of podocytopathy [35, 36]. Dysregulation of the alternative complement pathway can cause immune complex-mediated glomerulonephritis and C3 glomerulopathy [37], while viral infections such as hepatitis C virus and human immunodeficiency virus are associated with several glomerular diseases [38]. Monoclonal gammopathies should also be considered, as monoclonal proteins can produce a broad spectrum of glomerular lesions [39]. A practical disease-specific approach to the investigation of secondary causes, including recommended laboratory tests, serologic markers, genetic evaluation, and malignancy screening strategies, is summarized in Fig. 2. When a secondary cause is identified, management should primarily target the underlying disorder.

### P4 — Supportive and prophylactic management

#### Monitor blood pressure, salt intake, lipid profile, and edema regularly. Start prophylactic anticoagulation in patients with MN or persistent nephrosis at high risk of thromboembolic events.

Supportive care should remain the backbone of the management of NS. Renin–angiotensin system (RAS) inhibitors should be used to reduce proteinuria. Sodium–glucose co-transporter 2 inhibitors are generally safe and are now considered important antiproteinuric agents in patients with glomerular diseases [40]. Notably, a certain percentage of patients with MN at low to moderate risk can achieve remission without immunosuppressive agents [13]. Blood pressure control will not only delay progressive loss of kidney function but also protect against cardiovascular risks. Sodium intake should be limited to 2 g/day, and diuretics remain the cornerstone of edema management [13]. However, nephrotic syndrome is frequently complicated by diuretic resistance due to altered tubular handling of loop diuretics, enhanced distal sodium reabsorption, and variable intravascular volume status [41]. In patients with inadequate response to oral loop diuretics, intravenous administration, sequential nephron blockade with the addition of a thiazide-type diuretic, or amiloride may be considered [42]. Albumin co-infusion has also been used in selected cases, although supporting evidence remains limited [43]. Treatment of hyperlipidemia should be considered, especially in patients with other cardiovascular risk factors such as diabetes and hypertension [1, 13]. However, it should be acknowledged that no randomized controlled trial has specifically evaluated lipid-lowering therapy for cardiovascular outcomes in nephrotic syndrome. Current recommendations are largely extrapolated from studies in chronic kidney disease and supported by expert opinion. Notably, all of these mentioned supportive measures might not be needed in cases with steroid-sensitive podocytopathies, which tend to go into complete remission in a few weeks.

Thromboembolism is a common complication of NS, particularly MN [44]. Histologic type of injury, proteinuria, and serum albumin levels predict the risk of thrombosis, and hypoalbuminemia remains the most significant single predictor [13]. Risk is very high in patients with serum albumin levels ≤2.5 g/dl (measured with bromocresol green). Bromocresol purple gives systematically lower values and the anticoagulation threshold should be adjusted according to the assay used [13]. We believe that prophylactic anticoagulation should be administered in these cases. However, bleeding risk should be considered, and there is still no randomized trial data confirming the efficacy and safety of prophylactic anticoagulation in this setting. Although direct oral anticoagulants are increasingly used in clinical practice, uncertainties remain regarding their pharmacokinetics in severe nephrotic syndrome because of altered protein binding and urinary protein losses [45]. Ongoing studies are expected to provide further evidence. Until more robust data become available, warfarin remains the anticoagulant with the greatest clinical experience in patients with severe hypoalbuminemia.

### P5 — Specific IST

#### Use glucocorticoids as first-line treatment for primary podocytopathies. Consider B-cell-depleting agents or calcineurin inhibitors in relapsing or steroid-dependent/resistant cases. For primary MN, use rituximab in patients without high risk of progression; reserve cyclophosphamide plus glucocorticoids for those with severe kidney function impairment or life-threatening complications. Newer B-cell-depleting agents are suggested in resistant patients with primary podocytopathies and MN.

Primary podocytopathies are generally responsive to glucocorticoids. Notably, the initial response to treatment also provides prognostic information [10]. Resistance to glucocorticoids is quite common in FSGS, occurring in 40%–60% of patients and associated with a poor prognosis [46]. In steroid-dependent or frequently relapsing NS, rituximab or calcineurin inhibitors are useful to avoid prolonged glucocorticoid use, and rituximab also reduces relapse rates [10, 47, 48]. The benefits of rituximab are usually diminished in patients with glucocorticoid resistance [49], but calcineurin inhibitors can be helpful in these cases [13]. It should be kept in mind that unresponsive patients may not have true primary podocytopathies, and genetic evaluation should be considered (please see N1 below) [33].

Rituximab is the treatment of choice for patients with MN who have low to moderate risk [13, 50] and are mostly positive for anti-PLA2R antibodies. According to the KDIGO 2021 guideline [13], risk assessment is based on an integrated evaluation of proteinuria severity and persistence, eGFR, serum albumin levels, anti-PLA2R antibody titers and their evolution over time, and complications related to nephrotic syndrome. Patients with preserved kidney function, low anti-PLA2R antibody levels (<50 RU/ml), and improving proteinuria over the first 6 months are considered at lower risk, whereas declining eGFR, high or increasing anti-PLA2R antibody titers, or severe nephrotic syndrome-related complications indicate higher risk. Moderate risk is defined as the presence of persisting nephrotic-range proteinuria for more than 6 months in patients without the high-risk criteria. Calcineurin inhibitors with or without low-dose glucocorticoids can also be added to the regimen in patients with moderate risk. Cyclophosphamide plus glucocorticoids is useful in patients with high to very high risk of progression, particularly those with deteriorating kidney function, severe nephrotic syndrome, or other serious disease-related complications, and lower doses may be considered due to safety concerns [51]. Notably, obinutuzumab, a humanized glycoengineered type II anti-CD20 monoclonal antibody, may be useful in refractory primary podocytopathies and MN, and data suggesting its potential use as a first-line agent are accumulating [52–55]. It has been demonstrated that obinutuzumab achieves better B-cell clearance than rituximab, thereby inducing more durable responses [56, 57]. In patients who remain refractory to both rituximab and cyclophosphamide-based regimens, emerging plasma-cell-targeted therapies may represent a promising option. Anti-CD38 monoclonal antibodies such as daratumumab and felzartamab have shown encouraging results in refractory MN by targeting long-lived plasma cells that continue to produce pathogenic antibodies despite B-cell depletion [58].

Management of nephrotic syndrome during pregnancy requires special consideration because several standard therapies may be contraindicated. Renin–angiotensin system inhibitors should be discontinued due to potential fetal toxicity, and IST should be individualized, avoiding agents with known teratogenic effects. Given the increased risk of thromboembolic events associated with both nephrotic syndrome and pregnancy, thromboprophylaxis should be considered on a case-by-case basis. Close maternal and fetal monitoring, including assessment of kidney function, proteinuria, blood pressure, and fetal growth, is essential, and management should ideally involve a multidisciplinary team [59].

### N1 — Do not delay genetic testing

#### Do not postpone genetic testing when remission is not achieved.

Establishing a molecular genetic diagnosis is a critical step in the management of NS, as identifying a genetic cause can help avoid ineffective and potentially harmful IST. Although the prevalence of identifiable genetic disease declines with age, recent evidence shows that up to 10%–22% of adult-onset cases of FSGS also have a genetic basis, making testing relevant across all age groups [60, 61]. While many complex diseases are polygenic, most genetic forms of steroid-resistant NS (SRNS) and FSGS are monogenic, caused by rare variants with high effect sizes. However, genome-wide association studies are beginning to identify common variants that influence susceptibility, suggesting a more complex genetic architecture [61–63].

Adult-onset genetic NS more commonly follows an autosomal dominant pattern, typically lacks syndromic extrarenal features, and is largely unresponsive to IST [64]. An important exception is familial Mediterranean fever, a rare autoinflammatory disease caused by mutations in the *MEFV* gene and inherited in an autosomal recessive pattern, which may lead to NS because of secondary amyloidosis. A positive family history of NS or chronic kidney disease (CKD) further supports the possibility of an underlying genetic etiology. In addition, persistent microhematuria with dysmorphic red blood cells may point to collagen-IV-related disorders (*COL4A3–5*), which can present with an FSGS pattern on kidney biopsy [65]. Genetic testing should be considered in patients with SRNS who fail to achieve remission after 8–16 weeks of adequate steroid treatment [66], and in SRNS patients being evaluated for transplantation to help estimate the risk of disease recurrence.

Key genes implicated in adults include podocin (*NPHS2*) (often the R229Q variant in compound heterozygosity), Transient Receptor Potential Cation Channel Subfamily C 6 (*TRPC6*) (dominant gain-of-function variants with high risk of end stage kidney disease), Actinin Alpha 4 (*ACTN4*) (disrupted actin dynamics), and in rarer causes, such as Anillin, Actin Binding Protein (*ANLN*), and Podocalyxin-like (*PODXL*), which impair podocyte structure and function [64].

A genetic diagnosis has significant implications for the risk of post-transplant recurrence. While primary FSGS has a high recurrence rate, many monogenic forms show lower risks. For example, early reports for *ANLN* and *PODXL* mutations showed no recurrence in the kidney allograft following transplantation [67, 68]. Genetic testing is also important when evaluating living-related donors, as it helps avoid donation from asymptomatic mutation carriers.

Despite the clinical value, genetic testing in adult SRNS remains underutilized, largely driven by family history or unexplained CKD and is not guided by formal adult-specific recommendations.

### N2 — Do not prolong ineffective immunosuppression

#### Avoid continuing IST without clinical response in order to minimize toxicity and infection risk.

Prolonging ineffective IST exposes patients to cumulative toxicity without improving outcomes and should be avoided. The most used agents carry substantial dose- and duration-dependent risks. Glucocorticoids cause broad systemic toxicity, including increased risk of infections, metabolic and cardiovascular complications, osteoporosis, avascular necrosis, and adrenal suppression [69]. Cyclophosphamide carries dose-dependent risks including cytopenias, infertility, hemorrhagic cystitis, and malignancy, and its cumulative exposure should be carefully tracked against generally accepted safety thresholds of 36 g for oral and 10–12 g for intravenous administration [69–71]. Calcineurin inhibitors are primarily limited by nephrotoxicity and metabolic effects [72], as well as significant drug interactions, while rituximab, although generally better tolerated, may still lead to infusion reactions, an increased risk of infections, hypogammaglobulinemia, and late-onset neutropenia [69]. Continuing these therapies without clear benefit, therefore, adds harm rather than efficacy.

A structured, treatment-response-guided approach is essential. Immunological biomarkers can provide early evidence of treatment efficacy: in PLA2R-positive MN, declining and/or disappearing antibody titers precede clinical remission and support tapering, whereas persistently elevated titers indicate resistance and should prompt a change in therapy [73]. With improved assay standardization, anti-nephrin antibodies may potentially serve as biomarkers of disease activity in podocytopathies [27]. Reassessment at 3–6 months, based on proteinuria, kidney function, and biomarker levels, is recommended to determine whether treatment should be continued, modified, or stopped [73].

For rituximab, monitoring both B-cell depletion and immunoglobulin levels provides a practical window into treatment efficacy and safety. Depletion is assessed by flow cytometry measuring CD19+ or CD20+ B cells in peripheral blood. Importantly, peripheral counts do not reliably reflect tissue-level clearance in lymph nodes or spleen, a distinction relevant to obinutuzumab’s proposed deeper tissue penetration [74]. B-cell recovery typically occurs at 6–12 months after standard dosing but may be prolonged with repeated courses, and serial CD19 monitoring therefore informs re-treatment timing [75]. Concurrent immunoglobulin surveillance allows confirmation of therapeutic response and early detection of treatment-related hypogammaglobulinemia [69]. When response is inadequate, early transition to alternative or targeted therapies is preferable to escalating ineffective regimens.

### N3 — Do not miss post-transplant monitoring

#### Do not neglect post-transplant surveillance for early recurrence in patients with primary NS.

Recurrent glomerulonephritis is a significant, yet frequently under-recognized, cause of kidney allograft failure, accounting for ∼15%–22% of death-censored graft losses [76]. A major challenge in post-transplant care is that recurrence may remain clinically silent or with atypical presentation for extended periods, with overt signs such as proteinuria or declining kidney function emerging only after substantial injury has already occurred. Consequently, relying solely on clinical triggers to perform graft biopsy is often insufficient. In recurrent FSGS, histologic injury has been shown to precede clinical manifestations by 10 days to 2 months [77], underscoring the value of protocol biopsies, which can detect recurrence earlier and more frequently than clinically indicated biopsies.

To avoid missing the window for effective intervention, regular surveillance is essential. Risk stratification further refines follow-up intensity. Genetic or monogenic NS rarely recurs, whereas idiopathic or immune-mediated forms carry the highest risk. Especially, a subset of patients with idiopathic NS can have circulating disease drivers, and they experience dramatic disease recurrence after transplantation [78]. For patients with a history of primary FSGS, consensus guidelines recommend monitoring proteinuria and serum creatinine daily for the first week, weekly for the first month, and monthly for the remainder of the first year [77]. Biomarkers may also assist early detection: serial monitoring of anti-PLA2R antibody titers both before and after transplantation is vital, as high pre-transplant levels are strongly predictive of early recurrence [79]. Recent research has identified anti-nephrin autoantibodies as a significant candidate for the elusive “circulating factor” that drives the recurrence of primary podocytopathies after kidney transplantation [30, 80].

The goal of rigorous monitoring is early detection, thereby allowing timely intervention to prevent irreversible injury and avoid graft loss. Therapies such as rituximab or intensive plasma exchange have been shown to achieve high remission rates in recurrent disease and significantly improve allograft survival [81]. Novel targeted treatments may also include anti-CD38 antibodies [82].

### N4 — Do not ignore cardiovascular risk

#### Do not overlook cardiovascular risk factors such as persistent hypoalbuminemia, dyslipidemia, and reduced eGFR. Cardiovascular assessment should be integrated into long-term care.

NS is far more than a kidney-limited condition; it is a systemic disease that places patients at substantial cardiovascular risk. Clinical data show up to a two-fold increase in myocardial infarction and stroke, and nearly an eight-fold increase in venous thromboembolism, particularly in MN [83]. These complications are not rare events, but a central part of the disease burden.

The cardiovascular profile also varies by underlying cause. Patients with FSGS have the highest adjusted risk of coronary artery disease and heart failure, whereas those with MN are especially vulnerable to venous thromboembolism, largely due to severe hypoalbuminemia [1]. This excess risk reflects several interacting mechanisms, including an atherogenic lipid pattern with elevated low-density lipoprotein cholesterol and lipoprotein(a), a hypercoagulable state caused by urinary loss of anticoagulant proteins such as antithrombin III, and endothelial dysfunction driven by systemic inflammation [84].

Traditional cardiovascular risk scores, such as the Framingham, QRISK3, or the systematic coronary risk evaluation 2 (SCORE2) models, often underestimate true risk in NS, because they do not account for disease-specific factors such as proteinuria or low serum albumin. This can lead to under-recognition of cardiovascular morbidities.

Management must therefore extend beyond kidney-focused care. Early RAS inhibition, appropriate statin therapy, particularly in patients over 50 years of age, and consideration of prophylactic anticoagulation in patients with severe hypoalbuminemia are essential components of comprehensive care. Proactive attention to these systemic risks may prevent serious cardiovascular events long before kidney failure occurs.

### N5 — Do not overlook preventable infectious complications in patients receiving IST

#### Screen for latent infections and give antimicrobial prophylaxis, if indicated.

Infectious complications continue to be a major cause of morbidity and mortality in patients with NS treated with IST, yet they are still too often underestimated or detected only after significant harm has occurred. The risk is not driven solely by treatment. It reflects the combined burden of the nephrotic state, reduced kidney function, and therapy-induced immunosuppression. Urinary loss of immunoglobulins and complement factors, impaired cellular immunity, and edema-related skin fragility create an inherent vulnerability that is further amplified by glucocorticoids, cytotoxic agents, calcineurin inhibitors, and B-cell-depleting therapies [2].

Observational data consistently show higher infection rates in patients receiving IST than in those receiving conservative management, with the lungs, skin, and urinary tract most frequently involved. Patients with serum albumin levels below 2.5 g/dl, advanced age, acute kidney injury, or an inadequate treatment response represent a particularly high-risk subgroup and require especially close clinical monitoring [85]. Importantly, many infectious events are preventable. Vaccination against influenza, *Streptococcus pneumoniae*, and varicella zoster is essential and should ideally be completed before starting IST to ensure an adequate immune response [2]. Hepatitis B virus (HBV) reactivation is more frequent with high-dose glucocorticoids and B-cell-depleting therapies, while tuberculosis reactivation is largely linked to glucocorticoids and is negligible with B-cell-targeting agents [2, 86, 87]. When screening is overlooked and antiviral or chemoprophylactic therapy is delayed or omitted, these reactivations can progress to severe, yet largely avoidable, disease.

All patients facing IST should be screened for HBV prior to treatment initiation, including HBsAg, anti-HBs, and anti-HBc; HBsAg-positive patients additionally require serum HBV DNA quantification to exclude active infection. Prophylactic antiviral therapy is compulsory in HBsAg-positive patients and in those who are HBsAg-negative but anti-HBc-positive before anti-CD20 therapy, and is also warranted when glucocorticoids exceed 20 mg prednisolone daily for more than 4 weeks. Preferred agents include tenofovir alafenamide, tenofovir disoproxil fumarate, or entecavir, with lamivudine reserved as a secondary option. Prophylaxis should be continued for at least 12 months after the last anti-CD20 dose, and input from hepatology or infectious disease is advisable in complex cases [2, 88].

Screening for latent tuberculosis infection (LTBI) should ideally be performed in endemic regions, before initiating IST using the tuberculin skin test and/or interferon-gamma release assay (IGRA). IGRA is preferred in vaccinated individuals, alongside a chest radiograph to exclude active disease. Ongoing IST reduces the sensitivity of both tests, reinforcing the value of early screening. Once LTBI is confirmed and active tuberculosis is excluded, rifamycin-based chemoprophylaxis (4 months of rifampicin or 3 months of weekly rifapentine–isoniazid) is preferred over isoniazid monotherapy because of its shorter duration and better tolerability. Prophylaxis should ideally be completed before starting IST, though at least 4 weeks of treatment prior to therapy initiation is generally acceptable [2, 89].

The concept of the “net state of immunosuppression” is central to clinical decision-making. Prolonged or combined regimens, prior cyclophosphamide exposure, leukopenia, and hypogammaglobulinemia increase infection risk beyond the effects of any single drug [2]. Rituximab warrants particular caution, as sustained B-cell depletion, impaired vaccine responsiveness, and secondary hypogammaglobulinemia may predispose patients to serious infections months after administration [90]. It should also be noted that hypogammaglobulinemia may persist or worsen in patients who remain nephrotic under anti-B-cell therapy, reflecting a combination of urinary losses and treatment effect [91]. IgG replacement therapy in secondary hypogammaglobulinemia is most clearly supported for patients with recurrent serious infections and severe hypogammaglobulinemia, with IgG consistently below 3 g/l, or without recurrent serious infections but with impaired vaccine responses [92, 93]. Whether replacement offers meaningful benefit in autoimmune diseases as primary prophylaxis in moderate hypogammaglobulinemia without overt infections remains uncertain.

Prevention must therefore be intentional and forward-looking. Systematic screening for latent infections, timely vaccination whenever feasible, targeted antimicrobial prophylaxis in high-risk settings, regular monitoring of leukocyte counts, and immunoglobulin levels are essential. Overlooking these measures can undermine otherwise successful disease control and expose patients to complications that are, in many cases, preventable.

## CONCLUSION

Nephrotic syndrome requires a vigilant clinical approach that integrates accurate diagnosis, appropriate disease-specific therapy, and comprehensive supportive care. Kidney biopsy, immunologic biomarkers, and timely genetic testing play important roles in clarifying the underlying etiology and guiding treatment decisions. At the same time, clinicians must remain attentive to frequently overlooked aspects of care, including prevention of thromboembolic, cardiovascular, and infectious complications, as well as early recognition of treatment resistance or post-transplant recurrence. Avoiding prolonged ineffective IST and reassessing therapeutic strategies when response is inadequate are equally important. We believe greater awareness of these practical considerations may support more careful and balanced management of patients with NS.

## Supplementary Material

sfag268_Supplemental_File Ten slides for each tip for the diagnosis and management of nephrotic syndrome.

## Data Availability

No new data were generated or analyzed in support of this research.

## References

[1] Untersulzner C, Gauckler P, Matyjek A et al. Cardiovascular disease and risk management in patients with nephrotic syndrome. Kidney Res Clin Pract. 2025;45:467–78. 10.23876/j.krcp.25.177 42298988 PMC13347090

[2] Windpessl M, Kostopoulou M, Conway R et al. Preventing infections in immunocompromised patients with kidney diseases: vaccines and antimicrobial prophylaxis. Nephrol Dial Transplant. 2023;38:ii40–i9. 10.1093/ndt/gfad080 37218705

[3] Rovin BH, Adler SG, Barratt J et al. KDIGO 2021 clinical practice guideline for the management of glomerular diseases. Kidney Int. 2021;100:S1–S276. 10.1016/j.kint.2021.05.021 34556256

[4] Schnuelle P . Renal biopsy for diagnosis in kidney disease: indication, technique, and safety. J Clin Med. 2023;12:6424. 10.3390/jcm12196424 37835066 PMC10573674

[5] Najafian B, Lusco MA, Alpers CE et al. Approach to kidney biopsy: core curriculum 2022. Am J Kidney Dis. 2022;80:119–31. 10.1053/j.ajkd.2021.08.024 35125261

[6] Nasr SH, Fidler ME, Said SM. Paraffin immunofluorescence: a valuable ancillary technique in renal pathology. Kidney Int Rep. 2018;3:1260–6. 10.1016/j.ekir.2018.07.008 30450452 PMC6224795

[7] Agrawal V, Sharma A. Diagnostic immunostaining of renal biopsies: an overview of markers for glomerular diseases. Glomerular Dis. 2025;5:176–90. 10.1159/000545311 40303503 PMC12040309

[8] Madden B, Singh RD, Haas M et al. Apolipoprotein e is enriched in dense deposits and is a marker for dense deposit disease in c3 glomerulopathy. Kidney Int. 2024;105:1077–87. 10.1016/j.kint.2024.02.013 38447879

[9] Hurdogan O, Mirioglu S, Dirim AB et al. Apolipoprotein e immunostaining has diagnostic utility in differentiating dense deposit disease and c3 glomerulonephritis: clone-based evaluation of d719n, ep1373y, and 1b2c9. Mod Pathol. 2025;38:100874. 10.1016/j.modpat.2025.100874 40865921

[10] Mirioglu S, Daniel-Fischer L, Berke I et al. Management of adult patients with podocytopathies: an update from the era immunonephrology working group. Nephrol Dial Transplant. 2024;39:569–80. 10.1093/ndt/gfae025 38341276 PMC11024823

[11] Ishizuka K, Miura K, Hashimoto T et al. Degree of foot process effacement in patients with genetic focal segmental glomerulosclerosis: a single-center analysis and review of the literature. Sci Rep. 2021;11:12008. 10.1038/s41598-021-91520-9 34103591 PMC8187362

[12] Navarro-Torres M, Wooden B, Santoriello D et al. Podocytopathies. Adv Kidney Dis Health. 2025;32:2–11. 10.1053/j.akdh.2024.10.004 40175026

[13] Kidney Disease: Improving Global Outcomes Glomerular diseases Work Group . KDIGO 2021 clinical practice guideline for the management of glomerular diseases. Kidney Int. 2021;100:S1–S276. 10.1016/j.kint.2021.05.021 34556256

[14] Trimarchi H, Barratt J, Cattran DC et al. Oxford classification of IgA nephropathy 2016: an update from the IgA nephropathy classification working group. Kidney Int. 2017;91:1014–21. 10.1016/j.kint.2017.02.003 28341274

[15] Barbour SJ, Coppo R, Zhang H et al. Evaluating a new international risk-prediction tool in IgA nephropathy. JAMA Intern Med. 2019;179:942–52. 10.1001/jamainternmed.2019.0600 30980653 PMC6583088

[16] Bomback AS, Santoriello D, Avasare RS et al. C3 glomerulonephritis and dense deposit disease share a similar disease course in a large united states cohort of patients with c3 glomerulopathy. Kidney Int. 2018;93:977–85. 10.1016/j.kint.2017.10.022 29310824

[17] Caravaca-Fontán F, Trujillo H, Alonso M et al. Validation of a histologic scoring index for c3 glomerulopathy. Am J Kidney Dis. 2021;77:684–695.e1. 10.1053/j.ajkd.2020.11.011 33359150

[18] Mirioglu S, Cebeci E, Yazici H et al. Prognostic factors and validation of the histologic chronicity score for c3 glomerulopathy: a registry analysis. Clin Kidney J. 2024;17:sfae077. 10.1093/ckj/sfae077 39421234 PMC11483614

[19] Bobart SA, Han H, Tehranian S et al. Noninvasive diagnosis of pla2r-associated membranous nephropathy: a validation study. Clin J Am Soc Nephrol. 2021;16:1833–9. 10.2215/CJN.05480421 34782349 PMC8729491

[20] Caza TN, Larsen CP. False-positive anti-pla2r ELISA testing in patients with diabetes mellitus. Kidney Int. 2023;103:425. 10.1016/j.kint.2022.11.004 36681457

[21] Sethi S, Beck LH, Glassock RJ et al. Mayo clinic consensus report on membranous nephropathy: proposal for a novel classification. Kidney Int. 2023;104:1092–102. 10.1016/j.kint.2023.06.032 37795587

[22] Mirioglu S, Bruchfeld A, Caravaca-Fontan F et al. Quo vadis standardization of anti-nephrin antibody detection?. Glomerular Dis. 2025;5:200–5. 10.1159/000545706 40337460 PMC12058110

[23] Beck LH, Bonegio RGB, Lambeau G et al. M-type phospholipase a2 receptor as target antigen in idiopathic membranous nephropathy. N Engl J Med. 2009;361:11–21. 10.1056/NEJMoa0810457 19571279 PMC2762083

[24] Lerner GB, Virmani S, Henderson JM et al. A conceptual framework linking immunology, pathology, and clinical features in primary membranous nephropathy. Kidney Int. 2021;100:289–300. 10.1016/j.kint.2021.03.028 33857571

[25] Caza TN, Arivett BA, Hassen SI et al. Detection and characterization of nell1 autoantibodies in nell1 positive membranous nephropathy. Kidney Int. 2025;108:427–32. 10.1016/j.kint.2025.05.025 40541723 PMC12705032

[26] Watts AJB, Keller KH, Lerner G et al. Discovery of autoantibodies targeting nephrin in minimal change disease supports a novel autoimmune etiology. J Am Soc Nephrol. 2022;33:238–52. 10.1681/ASN.2021060794 34732507 PMC8763186

[27] Hengel FE, Dehde S, Lassé M et al. Autoantibodies targeting nephrin in podocytopathies. N Engl J Med. 2024;391:422–33. 10.1056/NEJMoa2314471 38804512

[28] Raglianti V, Angelotti ML, De Chiara L et al. Anti-nephrin, anti-podocin and anti-kirrel1 antibodies: biological challenges and clinical implications. Nephrol Dial Transplant. 2025;41:428–36. 10.1093/ndt/gfaf156 40815258

[29] Kronbichler A, Barnini C, Matyjek A et al. Antibody-mediated podocytopathies: a disease entity that implies immunotherapy. Nephrol Dial Transplant. 2025;40:218–21. 10.1093/ndt/gfae166 39020250

[30] Shirai Y, Miura K, Morimoto K et al. Reevaluating anti-nephrin autoantibodies by ELISA using human embryonic kidney-derived recombinant extracellular domain of human nephrin. Kidney Int. 2025;107:757–9. 10.1016/j.kint.2025.01.003 40118589

[31] Batal I, Watts AJB, Gibier J-B et al. Pre-transplant anti-nephrin antibodies are specific predictors of recurrent diffuse podocytopathy in the kidney allograft. Kidney Int. 2024;106:749–52. 10.1016/j.kint.2024.07.022 39127225 PMC11416305

[32] Habbig S, Debiec H, Chedik M et al. Anti-nephrin antibodies guide living donor kidney transplantation in a pediatric patient with primary focal segmental glomerular sclerosis. Kidney Int. 2025;108:321–7. 10.1016/j.kint.2025.04.015 40383230

[33] Romagnani P, Tang SCW, Weins A et al. Podocytopathies. Nat Rev Dis Primers. 2025;11:87. 10.1038/s41572-025-00671-w 41381622

[34] Murt A, Berke I, Bruchfeld A et al. Malignancies and glomerulonephritis: when to suspect and when to screen?. Clin Kidney J. 2025;18:sfaf101. 10.1093/ckj/sfaf101 40352576 PMC12062743

[35] Audard V, Larousserie F, Grimbert P et al. Minimal change nephrotic syndrome and classical hodgkin’s lymphoma: report of 21 cases and review of the literature. Kidney Int. 2006;69:2251–60. 10.1038/sj.ki.5000341 16672913

[36] Ribas A, Puche A, Gimeno J et al. Podocytopathy in patients with monoclonal gammopathy: three patients and literature review. Clin Kidney J. 2022;15:417–24. 10.1093/ckj/sfab176 35211301 PMC8862048

[37] Smith RJH, Appel GB, Blom AM et al. C3 glomerulopathy—understanding a rare complement-driven renal disease. Nat Rev Nephrol. 2019;15:129–43. 10.1038/s41581-018-0107-2 30692664 PMC6876298

[38] Stokes MB, Chawla H, Brody RI et al. Immune complex glomerulonephritis in patients coinfected with human immunodeficiency virus and hepatitis c virus. Am J Kidney Dis. 1997;29:514–25. 10.1016/S0272-6386(97)90332-2 9100039

[39] Leung N, Bridoux F, Batuman V et al. The evaluation of monoclonal gammopathy of renal significance: a consensus report of the international kidney and monoclonal gammopathy research group. Nat Rev Nephrol. 2019;15:45–59. 10.1038/s41581-018-0077-4 30510265 PMC7136169

[40] Caravaca-Fontán F, Stevens K, Padrón M et al. Sodium-glucose cotransporter 2 inhibition in primary and secondary glomerulonephritis. Nephrol Dial Transplant. 2024;39:328–40. 10.1093/ndt/gfad175 37550217

[41] Hoorn EJ, Ellison DH. Diuretic resistance. Am J Kidney Dis. 2017;69:136–42. 10.1053/j.ajkd.2016.08.027 27814935 PMC5182087

[42] Knauf H, Mutschler E. Sequential nephron blockade breaks resistance to diuretics in edematous states. J Cardiovasc Pharmacol. 1997;29:367–72. 10.1097/00005344-199703000-00010 9125675

[43] Lee TH, Kuo G, Chang C-H et al. Diuretic effect of co-administration of furosemide and albumin in comparison to furosemide therapy alone: an updated systematic review and meta-analysis. PLoS ONE. 2021;16:e0260312. 10.1371/journal.pone.0260312 34851962 PMC8635380

[44] Lionaki S, Derebail VK, Hogan SL et al. Venous thromboembolism in patients with membranous nephropathy. Clin J Am Soc Nephrol. 2012;7:43–51. 10.2215/CJN.04250511 22076873 PMC3265338

[45] Derebail VK, Zhu J, Crawford ML et al. Pharmacokinetics and pharmacodynamics of apixaban in nephrotic syndrome: findings from a phase 1a trial. Am J Kidney Dis. 2023;81:373–6. 10.1053/j.ajkd.2022.09.011 36328100 PMC12038909

[46] Gauckler P, Shin JI, Alberici F et al. Rituximab in adult minimal change disease and focal segmental glomerulosclerosis—what is known and what is still unknown?. Autoimmun Rev. 2020;19:102671. 10.1016/j.autrev.2020.102671 32942039

[47] Gauckler P, Matyjek A, Kapsia S et al. Long-term outcomes of rituximab-treated adult patients with podocytopathies. J Am Soc Nephrol. 2025;36:668–78. 10.1681/ASN.0000000520 39431468 PMC11975237

[48] Isaka Y, Sakaguchi Y, Shinzawa M et al. Rituximab for relapsing nephrotic syndrome in adults: a randomized clinical trial. JAMA. 2025;334:2011–19. 10.1001/jama.2025.19316 41191364 PMC12590383

[49] Tedesco M, Mescia F, Pisani I et al. The role of rituximab in primary focal segmental glomerular sclerosis of the adult. Kidney Int Rep. 2022;7:1878–86. 10.1016/j.ekir.2022.05.024 35967114 PMC9366368

[50] Gauckler P, Shin JI, Alberici F et al. Rituximab in membranous nephropathy. Kidney Int Rep. 2021;6:881–93. 10.1016/j.ekir.2020.12.035 33912740 PMC8071613

[51] Ramachandran R, Chauhan P, Bharati J et al. Randomized controlled trial on the efficacy of reduced-dose cyclical corticosteroids and cyclophosphamide in primary membranous nephropathy. Kidney Int Rep. 2025;10:3785–95. 10.1016/j.ekir.2025.08.018 41278314 PMC12639806

[52] Lin Y, Pan Y, Han Q et al. Obinutuzumab may be an effective and safe option for adult minimal change disease and focal segmental glomerulosclerosis patients after multitarget therapy including rituximab. Am J Nephrol. 2025;56:1–10. 10.1159/000541972 39396511

[53] Sethi S, Kumar S, Lim K et al. Obinutuzumab is effective for the treatment of refractory membranous nephropathy. Kidney Int Rep. 2020;5:1515–8. 10.1016/j.ekir.2020.06.030 32954076 PMC7486170

[54] Su X, Wu B, Tie X et al. Obinutuzumab as initial or second-line therapy in patients with primary membranous nephropathy. Kidney Int Rep. 2024;9:2386–98. 10.1016/j.ekir.2024.05.004 39156138 PMC11328588

[55] Fervenza FC, Hou FF, Hao C-M et al. Obinutuzumab or tacrolimus in primary membranous nephropathy. N Engl J Med. 2026. 10.1056/NEJMoa2602678 42246654

[56] Hu X, Zhang M, Xu J et al. Comparison of obinutuzumab and rituximab for treating primary membranous nephropathy. Clin J Am Soc Nephrol. 2024;19:1594–602. 10.2215/CJN.0000000000000555 39207845 PMC11637703

[57] Reddy VKC, Isenberg DA, Cambridge G et al. Obinutuzumab induces superior b-cell cytotoxicity to rituximab in rheumatoid arthritis and systemic lupus erythematosus patient samples. Rheumatology. 2017;56:1227–37. 10.1093/rheumatology/kex067 28407142 PMC5808665

[58] Rojas-Rivera JE, Ortiz A, Fervenza FC. Novel treatments paradigms: membranous nephropathy. Kidney Int Rep. 2023;8:419–31. 10.1016/j.ekir.2022.12.011 36938069 PMC10014375

[59] Wiles K, Chappell L, Clark K et al. Clinical practice guideline on pregnancy and renal disease. BMC Nephrol. 2019;20:401. 10.1186/s12882-019-1560-2 31672135 PMC6822421

[60] Yao T, Udwan K, John R et al. Integration of genetic testing and pathology for the diagnosis of adults with fsgs. Clin J Am Soc Nephrol. 2019;14:213–23. 10.2215/CJN.08750718 30647093 PMC6390925

[61] Caparali EB, De Gregorio V, Barua M. Genetic causes of nephrotic syndrome and focal and segmental glomerulosclerosis. Adv Kidney Dis Health. 2024;31:309–16. 10.1053/j.akdh.2024.04.001 39084756

[62] Connaughton DM, Hildebrandt F. Personalized medicine in chronic kidney disease by detection of monogenic mutations. Nephrol Dial Transplant. 2019;35:390–7. 10.1093/ndt/gfz028 PMC7057531 30809662

[63] Becherucci F, Landini S, Palazzo V et al. A clinical workflow for cost-saving high-rate diagnosis of genetic kidney diseases. J Am Soc Nephrol. 2023;34:706–20. 10.1681/ASN.0000000000000076 36753701 PMC10103218

[64] Massengill S, Trachtman H. Genetic spectrum of nephrotic syndrome: impact of podocytopathy in adult life. Adv Chronic Kidney Dis. 2022;29:221–4. 10.1053/j.ackd.2022.04.005 36084968

[65] Pilco-Terán M, Shabaka A, Furlano M et al. Indications for genetic testing in adults with focal segmental glomerulosclerosis. Nefrología (Engl Ed). 2025;45:135–49. 10.1016/j.nefroe.2025.02.001 39952830

[66] Liu J, Wang W. Genetic basis of adult-onset nephrotic syndrome and focal segmental glomerulosclerosis. Front Med. 2017;11:333–9. 10.1007/s11684-017-0564-1 28776307

[67] Gbadegesin RA, Hall G, Adeyemo A et al. Mutations in the gene that encodes the f-actin binding protein anillin cause fsgs. J Am Soc Nephrol. 2014;25:1991–2002. 10.1681/ASN.2013090976 24676636 PMC4147982

[68] Marx D, Caillard S, Olagne J et al. Atypical focal segmental glomerulosclerosis associated with a new PODXL nonsense variant. Mol Genet Genomic Med. 2021;9:e1658. 10.1002/mgg3.1658 33780168 PMC8172202

[69] Jefferson JA . Complications of immunosuppression in glomerular disease. Clin J Am Soc Nephrol. 2018;13:1264–75. 10.2215/CJN.01920218 30042223 PMC6086710

[70] Heijl C, Harper L, Flossmann O et al. Incidence of malignancy in patients treated for antineutrophil cytoplasm antibody-associated vasculitis: follow-up data from European vasculitis study group clinical trials. Ann Rheum Dis. 2011;70:1415–21. 10.1136/ard.2010.145250 21616914

[71] Huong DL, Amoura Z, Duhaut P et al. Risk of ovarian failure and fertility after intravenous cyclophosphamide. A study in 84 patients. J Rheumatol. 2002;29:2571–6. 12465154

[72] Hoorn EJ, Walsh SB, McCormick JA et al. The calcineurin inhibitor tacrolimus activates the renal sodium chloride cotransporter to cause hypertension. Nat Med. 2011;17:1304–9. 10.1038/nm.2497 21963515 PMC3192268

[73] Ronco P, Beck L, Debiec H et al. Membranous nephropathy. Nat Rev Dis Primers. 2021;7:69. 10.1038/s41572-021-00303-z 34593809

[74] Tur C, Eckstein M, Bucci L et al. Effects of different b-cell-depleting strategies on the lymphatic tissue. Ann Rheum Dis. 2025;84:2065–74. 10.1016/j.ard.2025.06.2120 40651933

[75] Efe O, Sauvage G, Jeyabalan A et al. Persistent b cell depletion after rituximab for autoimmune and glomerular diseases: a case series. Kidney Int Rep. 2025;10:1441–9. 10.1016/j.ekir.2025.02.002 40485690 PMC12142800

[76] Cosio FG, Cattran DC. Recent advances in our understanding of recurrent primary glomerulonephritis after kidney transplantation. Kidney Int. 2017;91:304–14. 10.1016/j.kint.2016.08.030 27837947

[77] Raina R, Jothi S, Haffner D et al. Post-transplant recurrence of focal segmental glomerular sclerosis: consensus statements. Kidney Int. 2024;105:450–63. 10.1016/j.kint.2023.10.017 38142038

[78] Hayward S, Parmesar K, Saleem MA. What is circulating factor disease and how is it currently explained?. Pediatr Nephrol. 2023;38:3513–8. 10.1007/s00467-023-05928-8 36952039 PMC10514121

[79] Hullekes F, Uffing A, Verhoeff R et al. Recurrence of membranous nephropathy after kidney transplantation: a multicenter retrospective cohort study. Am J Transplant. 2024;24:1016–26. 10.1016/j.ajt.2024.01.036 38341027

[80] Vivarelli M, Colucci M. Anti-nephrin antibodies in recurrence of focal segmental glomerulosclerosis: closer to discovering the holy grail?. Kidney Int. 2024;105:440–2. 10.1016/j.kint.2023.12.015 38388144

[81] Hickson LJ, Gera M, Amer H et al. Kidney transplantation for primary focal segmental glomerulosclerosis: outcomes and response to therapy for recurrence. Transplantation. 2009;87:1232–9. 10.1097/TP.0b013e31819f12be 19384172

[82] Cravedi P, Maggiore U, Mortari G et al. Combined anti-cd20/anti-cd38 therapy in posttransplant focal segmental glomerulosclerosis recurrence: a retrospective, international, multicenter study. Transplant Direct. 2026;12:e1908. 10.1097/TXD.0000000000001908 41567757 PMC12818861

[83] Go AS, Tan TC, Chertow GM et al. Primary nephrotic syndrome and risks of eskd, cardiovascular events, and death: the kaiser permanente nephrotic syndrome study. J Am Soc Nephrol. 2021;32:2303–14. 10.1681/ASN.2020111583 34362836 PMC8729848

[84] Parker K, Ragy O, Hamilton P et al. Thromboembolism in nephrotic syndrome: controversies and uncertainties. Res Pract Thromb Haemost. 2023;7:102162. 10.1016/j.rpth.2023.102162 37680313 PMC10480654

[85] Hsu C‐Y, Chang C, Chen H‐Y et al. Infectious complications in adult patients with idiopathic minimal change nephrotic syndrome undergoing immunosuppressive therapy. Nephrology. 2022;27:953–61. 10.1111/nep.14119 36209374

[86] Gameiro J, Windpessl M, Domingues P et al. Rituximab in glomerular diseases: indications, long-term use, complications and research gaps. Autoimmun Rev. 2026;25:103937. 10.1016/j.autrev.2025.103937 41072885

[87] Wang Xi, Hu S, Hong R et al. Risk of hepatitis b and tuberculosis reactivation in patients with neuromyelitis optica spectrum disorder undergoing b cell depletion therapy. Mult Scler Relat Disord. 2025;104:106850. 10.1016/j.msard.2025.106850 41240660

[88] Lau G, Yu M-L, Wong G et al. APASL clinical practice guideline on Hepatitis B reactivation related to the use of immunosuppressive therapy. Hepatol Int. 2021;15:1031–48. 10.1007/s12072-021-10239-x 34427860 PMC8382940

[89] Jha DK, Kakadiya R, Sharma A et al. Assessment and management for latent tuberculosis before advanced therapies for immune-mediated inflammatory diseases: a comprehensive review. Autoimmun Rev. 2025;24:103758. 10.1016/j.autrev.2025.103758 39870187

[90] Nixon A, Ogden L, Woywodt A et al. Infectious complications of rituximab therapy in renal disease. Clin Kidney J. 2017;10:455–60. 10.1093/ckj/sfx038 28852481 PMC5570071

[91] Otani IM, Lehman HK, Jongco AM et al. Practical guidance for the diagnosis and management of secondary hypogammaglobulinemia: a work group report of the aaaai primary immunodeficiency and altered immune response committees. J Allergy Clin Immunol. 2022;149:1525–60. 10.1016/j.jaci.2022.01.025 35176351

[92] Wijetilleka S, Mukhtyar C, Jayne D et al. Immunoglobulin replacement for secondary immunodeficiency after b-cell targeted therapies in autoimmune rheumatic disease: systematic literature review. Autoimmun Rev. 2019;18:535–41. 10.1016/j.autrev.2019.03.010 30844552

[93] Chung SA, Langford CA, Maz M et al. 2021 American college of rheumatology/vasculitis foundation guideline for the management of antineutrophil cytoplasmic antibody–associated vasculitis. Arthritis Rheumatol. 2021;73:1366–83. 10.1002/art.41773 34235894 PMC12327957

